# Polymyxins and Bacterial Membranes: A Review of Antibacterial Activity and Mechanisms of Resistance

**DOI:** 10.3390/membranes10080181

**Published:** 2020-08-08

**Authors:** Carole Ayoub Moubareck

**Affiliations:** College of Natural and Health Sciences, Zayed University, Dubai 19282, UAE; Carole.AyoubMoubareck@zu.ac.ae; Tel.: +971-4402-1745

**Keywords:** polymyxins, colistin, outer membrane, lipopolysaccharide, *mcr*, antibiotic resistance, Gram-negative pathogens

## Abstract

Following their initial discovery in the 1940s, polymyxin antibiotics fell into disfavor due to their potential clinical toxicity, especially nephrotoxicity. However, the dry antibiotic development pipeline, together with the rising global prevalence of infections caused by multidrug-resistant (MDR) Gram-negative bacteria have both rejuvenated clinical interest in these polypeptide antibiotics. Parallel to the revival of their use, investigations into the mechanisms of action and resistance to polymyxins have intensified. With an initial known effect on biological membranes, research has uncovered the detailed molecular and chemical interactions that polymyxins have with Gram-negative outer membranes and lipopolysaccharide structure. In addition, genetic and epidemiological studies have revealed the basis of resistance to these agents. Nowadays, resistance to polymyxins in MDR Gram-negative pathogens is well elucidated, with chromosomal as well as plasmid-encoded, transferrable pathways. The aims of the current review are to highlight the important chemical, microbiological, and pharmacological properties of polymyxins, to discuss their mechanistic effects on bacterial membranes, and to revise the current knowledge about Gram-negative acquired resistance to these agents. Finally, recent research, directed towards new perspectives for improving these old agents utilized in the 21st century, to combat drug-resistant pathogens, is summarized.

## 1. Introduction

The progressive global rise and dissemination of multidrug-resistant (MDR) bacteria represent an enormous threat to humans today, and a main concern to public health and modern health care systems [1]. Given their distinctive cellular features, Gram-negative bacteria are said to specifically develop and acquire dynamic resistance patterns and cause significant morbidity and mortality worldwide. Among these, carbapenem-resistant *Enterobacteriaceae*, MDR *Pseudomonas aeruginosa*, and MDR *Acinetobacter baumannii* represent serious pressures to antimicrobial therapy, due to the extremely limited efficacious treatment options [2]. Against such pathogens, polymyxins represent one of the last-resort antibiotics that is still effective [3], among few others, including fosfomycin, ceftazidime/avibactam and the recently approved meropenem–vaborbactam [4,5]. Polymyxins have recently been invigorated as a class of bactericidal drugs that disrupt the outer cell membrane, and with their revival, studies to understand their effects on bacterial cells were also actively restarted [6]. Nevertheless, the utility of polymyxins is currently facing a worldwide increasing resistance, particularly due to the plasmid-encoded mobilized colistin resistance (*mcr*) gene present in pathogens such as *Escherichia coli* and *Klebsiella pneumoniae* [7]. Polymyxins are definitely relevant in the context of biological membranes study, since the Gram-negative outer membrane represents their first and foremost target. In this paper, the main properties, mechanism of action, resistance pathways, and forthcoming clinical and research directions for polymyxins are reviewed.

## 2. Historical Background and Types of Polymyxins

**Figure 1:**
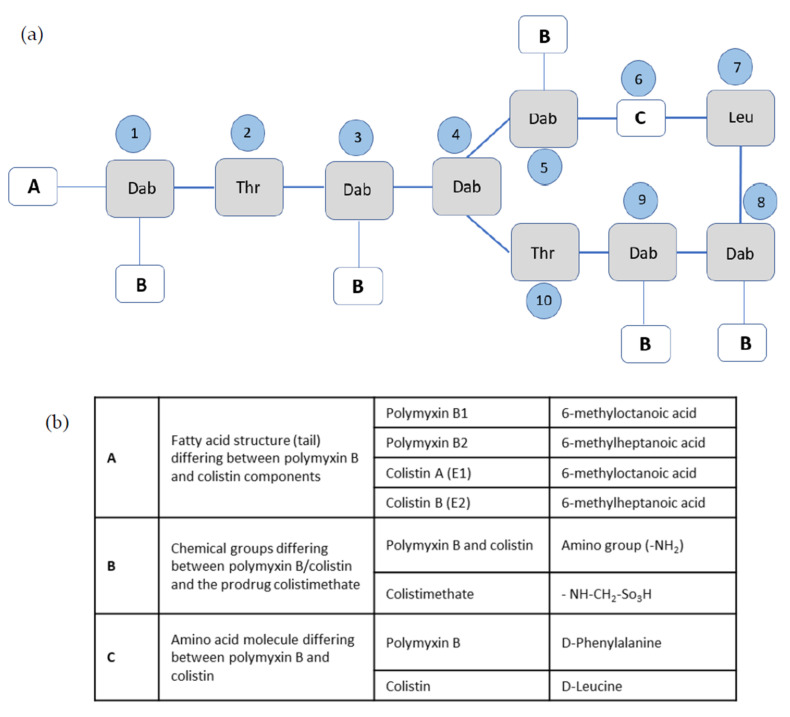
(**a**) Representation of the chemical structures of polymyxin B and colistin, with components B1, B2, E1, and E2, as well as colistimethate. The shaded boxes represent the fixed portions of the molecule, while the white boxes represent the structures that differ among polymyxin B, colistin, and/or colistimethate. The numbers in the blue circles correspond to the numbering of the amino acids from 1 to 10. The linear part of the molecule consists of a tripeptide (amino acids 1–3), while the cyclic part consists of a heptapeptide (amino acids 4–10). Dab = L-α,γ-diaminobutyric acid, Thr = Threonine; Leu = Leucine. (**b**) The groups A, B, and C in section (a) are described, and they represent the chemical moieties with variation between the polymyxins B1, B2, E1, E2 or between them and the prodrug colistimethate.

The polymyxins comprise a group of old antimicrobial substances that are nonribosomal cyclic lipopeptide, secondary metabolites of the soil, marine, and plant bacterium *Paenibacillus polymyxa* [8]. Formerly known as *Bacillus polymyxa* var. colistinus, this aerobic, Gram-positive, spore-forming organism was the source of colistin (also known as polymyxin E), originally discovered in 1947 by Benedict and Langlykke [9]. Four additional polymyxins, named A to D, were recovered from the metabolism fluid of different strains of *P. polymyxa* [10,11]. Polymyxin A was formerly called “Aerosporin” and polymyxin D “Polymyxin.” All polymyxins are cationic decapeptides, consisting of a cyclic heptapeptide linked to a linear tripeptide side chain acylated at the N terminus by a fatty acid tail [12]. A common property of polymyxins is including L-α,γ-diaminobutyric acid (Dab), the amino acid threonine, and a branched fatty acid in their structure. They differ in their amino acid composition, where all except polymyxin C contain leucine, polymyxins B and C contain phenylalanine, and only polymyxin D contains serine [11,12]. The structures of polymyxin B and colistin are shown and compared in Figure 1. The differences between the various polymyxin components, namely polymyxins B1, B2, E1, and E2 and colistimethate, are also shown and described shortly (Section 3).

Of all five polymyxins, only polymyxin B and colistin were put into clinical use in the 1950s, as they were found to be the least nephrotoxic. Eventually, these antibiotics fell out of favor and their systemic use waned due to their significant adverse effects, especially their potential for nephrotoxicity and neurotoxicity [12,13]. Early medicinal chemistry efforts with polymyxins in the 1970s aimed at modifying their structures to improve their safety. However, the majority of these trials were limited to modifications to the peripheries of the polymyxin structure, namely the acylation/alkylation of the side-chain amino groups of the Dab residue, or the substitution of the N-terminal fatty acyl chain [8]. At that time, the lack of robust synthetic platforms that allow the total synthesis of polymyxin lipopeptides, as well as the limited understanding of polymyxin pharmacology, were a barrier to obtain more tolerable and effective compounds [13], and their clinical use dramatically declined [14]. This was concurrent with the emergence of less-toxic aminoglycosides and other antipseudomonal agents [15]. Nevertheless, interest in polymyxin B and colistin has risen substantially in the last two decades, with the emergence of MDR Gram-negative organisms nonresponsive to other clinically available antibiotics [16,17]. In this review, and unless otherwise indicated, the term polymyxins shall be used to refer to the two clinically useful compounds, polymyxin B and colistin.

## 3. Clinically Useful Polymyxins and Their Principle Properties

Despite minor differences, polymyxin B and colistin have comparable chemical structures, mechanisms of action, and spectra of activity. However, they have different pharmacokinetics and pharmacodynamics. The sections below address the essential properties of the two clinically useful compounds.

### 3.1. Chemical Properties and Structure–Activity Relationships

Colistin and polymyxin B differ by a single amino acid at position 6 in the peptide ring, as shown in Figure 1, with a D-phenylalanine in polymyxin B and a D-leucine in colistin [18]. Both drugs are highly basic due to five free amino groups [19]. Polymyxin B is primarily composed of a mixture of two polypeptides, polymyxin B1 and polymyxin B2; colistin is primarily composed of a mixture of colistin A (also known as polymyxin E1) and colistin B (also known as polymyxin E2). The difference between these four compounds is the fatty acid substitution of the lipopeptide structure, where it consists of 6-methyloctanoic acid in polymyxins B1 and E1, and of 6-methylheptanoic acid in polymyxins B2 and E2 (Figure 1) [20]. 

Although the mechanistic action of polymyxin B and colistin shall be deliberated exquisitely later in this review, it is important to mention, in context of their structure, that their chemistry is critical to their antibacterial activity [18]. The primary amines of the Dab residues are ionized at physiological pH, allowing the polymyxin molecules to carry a net polycationic charge, a vital characteristic for their interaction with negatively charged phosphate groups of the lipid A of bacterial lipopolysaccharide (LPS). In addition, polymyxins possess hydrophobic domains, namely the fatty acyl side chain, and these are able to interact with corresponding LPS structures [21]. Therefore, as a result of such assembly of hydrophilic and lipophilic entities, polymyxins are amphipathic and capable of both ionic and hydrophobic molecular interactions at the bacterial outer membrane level, contributing to their mechanism of action [13]. Studies from models using monolayers of LPS of Gram-negative bacteria indicate that chemical cationic amphipathicity determines polymyxin activity [22].

A deep understanding of the structure–activity relationships of polymyxins has been established, including the capacity of their three-dimensional structure to interact with bacterial membranes [23]. Polymyxins initially act by binding to lipid A of LPS, whose anionic nature facilitates the electrostatic interaction with the cationic polymyxins. This primary interaction leads to the disruption of bacterial outer membrane and the hydrophobic insertion of the fatty acyl chain of polymyxin into lipid A. Subsequently, cytoplasmic membrane disruption and the potential additional intracellular interactions lead to cell death [24]. Important contributions were made by the N-terminal fatty acyl chain of polymyxins to binding with LPS and consequent antibacterial activity. A comparison across different N-terminal fatty acyl chains documented that antimicrobial activity correlated with the length and bulkiness of the N-terminal fatty acid, where seven to nine carbon atoms are optimal for binding affinity [13]. 

Another important structural feature of the polymyxins, vital for their activity, is the presence of multiple positively charged Dab side chains, which interact with the phosphate groups on lipid A [8]. The key features of the Dab residues that are important for LPS binding affinity include the cationic character, the two methylene groups of the Dab side chain, and the characteristic order of the Dab residues within the primary polymyxin sequence that confers the proper spatial distribution of the positive charges for electrostatic interactions with the phosphates of lipid A. Overall, the Dab residues, particularly those lying within the cyclic heptapeptide, are indispensable for antimicrobial activity [13,25]. Furthermore, polymyxins contain D-phenylalanine and L-leucine at the amino acid positions 6 and 7, respectively, in the fatty acid chain of polymyxin B, and D-leucine and L-leucine at positions 6 and 7, respectively, in colistin, within the heptapeptide ring. These amino acids ensure a hydrophobic motif believed to insert into the bacterial outer membrane and stabilize LPS complexation, via hydrophobic interactions with the fatty acyl chains of lipid A [26]. Finally, the particular combination of topographic chemical features and the special configuration of the polymyxin ring structure appears ideal for efficient binding to LPS and the subsequent membrane permeabilizing effect. Previous attempts to produce an extended ring structure, generated by the insertion of additional Dab residues, yielded resultant compounds that were significantly less active than polymyxins [27].

### 3.2. Spectrum of Antibacterial Activity

Polymyxins demonstrate important activity against Gram-negative aerobic pathogens, including most members of the *Enterobacteriaceae* family, *E. coli*, *Enterobacter* spp., *Klebsiella* spp., *Citrobacter* spp., *Salmonella* spp., and *Shigella* spp [12]. Additionally, they are effective against non-fermentative Gram-negative common pathogens, including *A. baumannii* and *P. aeruginosa*. The breakpoints of sensitivity for *Enterobacteriaceae*, *Pseudomonas*, and *Acinetobacter* according to the 2020 internationally adopted guidelines are shown in Table 1 [28,29]. These breakpoints correspond to the minimum inhibitory concentration (MIC) used to categorize strains as sensitive, resistant, or intermediate in their response to polymyxins. In vitro susceptibility was reported for *Stenotrophomonas maltophilia* [30]. Notable exceptions of Gram-negative bacteria that are naturally resistant to polymyxins comprise *Pseudomonas mallei*, *Morganella morganii*, *Vibrio cholerae*, *Serratia marcescens*, *Proteus* spp., *Providencia* spp., *Burkholderia cepacia*, *Chromobacterium* spp., *Edwardsiella* spp., *Brucella*, *Legionella*, and *Campylobacter*. Polymyxins are not active against Gram-negative cocci (such as *Neisseria* spp.), nor against Gram-positive and anaerobic bacteria, parasites or fungi [31].

Modifications in lipid A of LPS are associated with the innate resistance seen in bacteria intrinsically resistant to polymyxins [32]. For example, in Proteus mirabilis, the expression of a seven-gene operon, called the *pmrHFIJKLM* homologue, is involved in LPS modification, leading to polymyxin resistance [33]. In *Brucella melitensis*, lowering the phosphoethanolamine (pEtN) content of the cell envelope, using the enzyme BveA, a type of phospholipase, increases *B. melitensis* resistance to polymyxin B [34]. Conversely, in *Campylobacter jejuni*, modifying lipid A by the addition of pEtN, using the catalytic action of the enzyme pEtN transferase, promotes polymyxin resistance [35]. Nevertheless, recent research on the in vitro activity of colistin against *C. jejuni* and *Campylobacter coli*, obtained from fecal material of patients with severe diarrhea, have shown low inhibitory values [36]. The question whether colistin may represent an alternative to fluoroquinolones and tetracyclines for the treatment of severe diarrhea produced by these species warrants additional investigation. 

### 3.3. Administration and Clinical Uses

Polymyxin B and colistin harbor different modes of administration to maintain activity [37]. Polymyxin B is administered as the active antibacterial compound, polymyxin B sulfate, which is given intravenously. Polymyxin B is not absorbed from the gastrointestinal tract. Topical, nonabsorbable oral, and ophthalmic formulations are available. Additional routes of administration include inhaled and intrathecal administration [38]. 

In contrast, colistin is administered parenterally as a prodrug, colistin methanesulfonate, also named colistimethate sodium (CMS), which is considered less toxic than the parent drug, colistin sulfate, upon parenteral administration [39]. CMS itself lacks intrinsic antibacterial activity; it should be converted in vivo into colistin after its administration [18,40]. In chemical synthesis, CMS is produced via an interaction of colistin with formaldehyde and sodium bisulfite, leading to a sulfomethyl group being added to the primary amines of colistin [41]. As such, colistin and CMS also differ in administration: Colistin is primarily used topically, whereas CMS is used parenterally, and both may be given by inhalation [42]. 

#### 3.3.1. Systemic Use

Due to their poor oral absorption, orally administered polymyxins are only used for digestive tract disinfection. Moreover, polymyxins do not efficiently diffuse into tissues, neither do they penetrate the cerebrospinal fluid, nor the pleural and peritoneal cavities [43]. Nevertheless, polymyxins are used systemically by intravenous administration for serious infections due to pathogens resistant to other effective therapies. They are indicated for the treatment of diverse infections, including pneumonia, bacteremia, urinary tract infections, bone and joint infections, burn infections, endocarditis, cellulitis, cystic fibrosis, gynecologic infections, meningitis, and ventriculitis [44]. Whether polymyxin B or colistin are preferable for systemic administration is debatable. Some trials suggest that polymyxin B achieves adequate drug levels more rapidly and reliably than colistin, the latter being a prodrug. An exception may be infections of the urinary tract, where colistin appears to be more effective and reaches high urinary levels, probably due to the extrarenal clearance of polymyxin B, keeping its urinary concentrations low [18,45]. Inhaled forms of both polymyxin B and colistin are available, and are given via a nebulizer to reach the lungs for the management of chronic pneumonia caused by P. aeruginosa in cystic fibrosis patients. However, because polymyxin B is more likely to cause airway obstruction, colistin is generally preferred for this indication [46]. The intrathecal and intraventricular administration of either polymyxin B or colistin is useful for central nervous system infections due to MDR Gram-negative bacteria, as an adjunct to systemic antibiotic therapy. As for the inhaled route, clinical experience, safety, and efficacy appear to be higher for intrathecal and intraventricular colistin, while experience with polymyxin B via these routes is limited [47].

#### 3.3.2. Topical Use

Polymyxin B sulfate is available for ophthalmic, otic, and topical use in combination with a variety of other compounds, while colistin is available as otic drops [48]. Polymyxin B is used with bacitracin as an opthalmic ointment, while it is available with neomycin as a urinary bladder irrigant for short-term use (up to 10 days) in abacteriuric patients to help prevent bacteriuria and Gram-negative rod septicemia associated with the use of indwelling catheters. It is also available with both bacitracin and neomycin as a topical antibiotic [49]. Infections of the skin, mucous membranes, eye, and ear due to sensitive microorganisms respond to the local application of polymyxin B in solution or ointment form. External otitis, frequently due to *Pseudomonas*, may also be cured by the topical use of the drug. *P. aeruginosa* is a common cause of infection of corneal ulcers; the local application or subconjunctival injection of polymyxin B is often curative [50].

### 3.4. Pharmacokinetics

Polymyxin B is administered directly in its active antibacterial form, and is subject to very extensive renal tubular reabsorption and thus primarily undergoes nonrenal clearance. Pharmacokinetic data on polymyxin B are limited. Over 95% of polymyxin B is cleared independently of the kidneys. Little is known about its extravascular distribution or penetration into other tissues, but it is thought to be similar to that of colistin, with generally poor penetration into the lungs, pleura, bones, and central nervous system [38].

Unlike polymyxin B, and following parenteral administration, about 20–25% of the dose of CMS prodrug is hydrolyzed in vivo into an active colistin entity, while a large proportion of the prodrug is eliminated mainly through the kidneys by glomerular filtration and tubular secretion [51]. CMS (but not colistin) is cleared renally; thus, the modification of the dose is generally required in patients with impaired renal function. Therefore, colistin concentrations resulting from the original CMS administration are low. In contrast to CMS, colistin is eliminated predominantly by nonrenal pathways because of its extensive renal tubular reabsorption. Although colistin is poorly excreted in urine, the urinary concentration of colistin may be relatively high after the administration of CMS, since the conversion of most colistin recovered in the urine is from the post-excretion hydrolysis of CMS into colistin within the urinary tract [12]. Colistin is tightly bound to membrane lipids of the cells in many body tissues, including the liver, lung, kidney, brain, heart, and muscles. The half life of colistin following IV administration is about 2–3 hours in subjects with normal renal function, and it is about 50% protein bound [37].

### 3.5. Toxicity

Perhaps the most common and clinically significant adverse effect of intravenous polymyxins is nephrotoxicity, which has an incidence of 50–60% in patients receiving polymyxin B or colistin [52], with expert opinion suggesting that the relative risk of nephrotoxicity is not significantly different among the two compounds [53]. Polymyxins have correlations with hematuria, proteinuria, oliguria, and acute kidney injury. Therefore, renal function should be monitored closely during administration, and the concurrent use of other nephrotoxic drugs should be avoided whenever possible [54]. The plasma concentrations of polymyxins associated with the increased risk of acute renal failure intersect with those required for antibacterial effect, rendering these drugs of narrow therapeutic index, a quantitative measurement of the relative safety of a drug that compares the amount that causes the therapeutic effect to the amount that causes toxicity [55]. The requirement for the conversion and recycling of polymyxins in the kidneys, as described above, makes the proximal tubular cells of the kidney the major location where polymyxins accumulate, while their concentrations are much lower in the liver, heart, lungs, spleen, and muscles [56]. Cell line investigations and in vivo preclinical models studying polymyxins toxicity on renal tubular cells suggest several cellular mechanisms. These include oxidative stress, apoptosis, cell cycle arrest, and autophagy [57]. The nephrotoxic potential of polymyxins is associated with their chemical structure. Attempts for the complete removal of the N-terminal fatty acyl group, considered a nephrotoxicity “hot-spot”, or to decrease its hydrophobicity, thereby reducing its uptake by renal tubular cells, produced derivatives with reduced nephrotoxicity. However, this occurred at the expense of significantly reduced antibacterial activity compared with polymyxin B and colistin [8].

Added to nephrotoxicity, hypersensitivity reactions, manifested as rash, pruritus, urticaria, and fever have been reported with the systemic use of polymyxins, [54], as well as neurotoxicity, seen in about 7% of patients. The latter is clinically characterized by symptoms of dizziness, visual disturbance, ataxia, vertigo, confusion, hallucinations, seizures, and facial and peripheral paresthesias. The use of other neurotoxic drugs concomitantly should be avoided [58]. It is believed that such manifestations result from the injury and death of neuronal cells, largely caused by reactive oxygen-induced oxidative stress and mitochondrial dysfunction, followed by apoptosis and autophagy [59,60]. Experimental trials for the scavenging of reactive species and halting apoptosis in cell lines using certain agents claimed to be neuroprotective, such as minocycline and rapamycin, are underway [59,61,62]. Finally, apart from the above adverse effects and in some reports, skin hyperpigmentation was documented with intravenous polymyxin B, and was associated with melanocyte activation and inflammatory process in the skin [63,64].

## 4. Mechanism of Action of Polymyxins and Proposed Interactions with Bacterial Membranes and Other Cellular Structures

### 4.1. Overview

The study of mechanistic pathways used by polymyxins to kill Gram-negative bacteria has been the subject of extensive research. The expanding interest in the use of these antibiotics with a focus on their mechanism of antibacterial activity, and the subsequently emerging resistance in Gram-negative bacteria, has been nicely described in previous reviews [12,65]. Even though the details of the antibacterial activity of these compounds are yet to be thoroughly understood, their initial interaction with bacterial membranes is indispensable [21]. In this context, polymyxins, basic peptides with a molecular weight of about 1000 Da, act as surface-active amphipathic agents or cationic detergents. They interact strongly with phospholipids and disrupt the structure of cell membranes. Specifically, polymyxins bind to LPS and phospholipids in the outer cell membrane of Gram-negative bacteria. They competitively displace divalent cations from the phosphate groups of membrane lipids, which leads to the destabilization of the outer cell membrane, the leakage of intracellular contents, and bacterial cell death [66]. 

**Figure 2:**
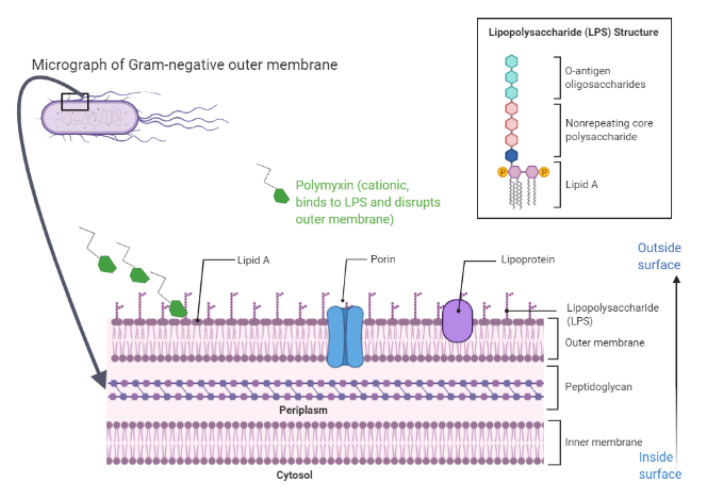
A schematic representation of a Gram-negative cell wall with outer membrane composition. The inner leaflet consists of phospholipids, whereas the outer leaflet shows lipopolysaccharides, lipoproteins and porins. Lipopolysaccharide components are also shown. Cationic polymyxins bind to the negatively charged components of lipopolysaccharides.

To properly comprehend the mechanism of action of polymyxins, an understanding of the outer membrane structure in Gram-negative bacteria is imperative. This membrane surrounds a thin peptidoglycan layer, and comprises an exclusive architecture, not found in Gram-positive bacteria [67]. Its structure allows it to be a potent selective permeability barrier against harmful molecules, such as detergents antibiotics [68]. The outer membrane organization and the binding of polymyxins are shown in Figure 2.

The chemical composition of the outer membrane is heterogeneous, with phospholipids, LPS, outer membrane proteins, and lipoproteins [69]. Such a blend of chemical components is arranged asymmetrically, where a bilayer of phospholipids, similar to other biological membranes, exists in the inner leaflet, and LPS in the outer leaflet, with anchored lipoproteins and outer membrane proteins [70]. Phospholipids of the outer membrane include different molecules, such as phophatidylethanolamine, phosphatidylglycerol, and cardiolipin [70]. Regarding the outer membrane anchored lipoproteins, these play a key role in the linkage between the outer membrane and peptidoglycan, peptidoglycan biosynthesis, flagellar assembly, and protein secretion and polysaccharide secretion [68,71]. Outer membrane proteins act as porins, specific and non-specific channels that regulate the transport of hydrophilic molecules across the outer membrane [72]. On the other hand, the LPS is a complex, glucosamine-based glycolipid unique to Gram-negative bacteria. It is composed of lipid A, core oligosaccharide, and O-antigen polysaccharide chains, and plays a critical role in the barrier function of the outer membrane [73]. Lipid A (endotoxin) is a powerful stimulator of human immune response, and is released upon bacterial death, resulting in the secretion of a number of proinflammatory cytokines from monocytes and macrophages, with the resulting possibility of Gram-negative sepsis [74,75]. The chemical structure of lipid A consists of D-glucosamine disaccharide that is phosphorylated at the 1′- and 4′ positions, with fatty acid esters attached to both carbohydrates. The fatty acyl chain length may vary between bacterial species, but is typically conserved within a given species. The lipid A chains are tightly packed together within the outer membrane through van der Waals forces, while divalent calcium and magnesium cations associated with lipid A act to bridge adjacent LPS molecules with each other [21,76]. Lipid A, the hydrophobic domain of LPS, lies on the outer membrane at the side of the phospholipid bilayer. Meanwhile, a non-repeating core oligosaccharide and a distal polysaccharide (also known as O-antigen or somatic-antigen) line the external portion of LPS, which extends to outside of the cell. Oligosaccharides are involved in growth, as well as in bacterial resistance to antibiotics, complement system, and various environmental stresses [77]. The resistance to stress is dependent on the negatively charged LPS, making the outer membrane impermeable to hydrophobic compounds, and on the outer membrane proteins folded with transmembrane domains. Some of these proteins form porins for the diffusion of small hydrophilic molecules. The outer membrane lipoproteins float among other components, giving the outer membrane an overall sophisticated composition, that is efficient to protect against toxic substances and stressful environmental conditions [78].

### 4.2. Insights into Models of Polymyxins’ Mechanism of Action

In fact, the exact mode of action of polymyxins lingers to be argumentative. Despite an overall assumption for the membrane as the primary drug target for polymyxins, there is evidence for alternative or complementary pathways, and literature has described several models. 

In one model based on outer membrane damage, consensus opinion focuses on a number of steps: (1) the initial uptake of polymyxin into the bacterial outer membrane is “self-promoted” [21]. In this process, polymyxins, with cationic affinity to LPS at least three-fold higher than that of the native divalent cations, calcium and magnesium, competitively displace these ions and, being bulky, disrupt the normal barrier property of the outer membrane. The affected outer membrane is thought to develop temporary “cracks”, that permit the passage of various molecules, among which is the uptake of the polymyxin itself [79]. (2) Following this uptake, an electrostatic interaction occurs between the Dab residue of the positively charged polymyxin on one side, and the phosphate groups of the negatively charged lipid A, making lipid A the principle polymyxin-binding target in the outer membrane of Gram-negative bacteria [13]. (3) Divalent calcium and magnesium cations are displaced from the negatively charged phosphate groups of membrane lipids [12], and this displacement allows the hydrophobic fatty acyl tail of the polymyxin molecule to be inserted into the outer membrane [79]. (4) Such insertion weakens the packing of adjacent lipid A fatty acyl chains, causing outer membrane expansion [13]. (5) Eventually, such expansion facilitates the formation of destabilized areas through which polymyxin crosses the outer membrane. Finally, polymyxins will destroy the physical integrity of the phospholipid bilayer of the cytoplasmic (inner) membrane, and intracellular contents begin to leak out, resulting in cell death [3,26].

Apart from such steps, and in other models, polymyxin is thought to mediate the fusion of the inner leaflets of the outer membrane and the outer leaflet of the inner membrane surrounding the periplasmic space [80]. Such action is believed to induce phospholipid exchange, between the leaflets of the inner and outer membranes, triggering a loss of specificity of phospholipid composition. This can potentially cause an osmotic imbalance that culminates in cell death [22]. Such a pathway, in which polymyxins bind to both anionic phospholipid vesicles, namely, inner phospholipid leaflets of outer membrane and inner membrane, promote phospholipid exchange between vesicles, is called the vesicle–vesicle contact pathway. The mechanism for the intermembrane transfer of phospholipids could be responsible for the intracellular trafficking and sorting of phospholipids; it could be a necessary step for polymyxin antibiotic action [81]. Moreover, hydrophobic interactions are assumed to occur between the N-terminal fatty acyl tail of the polymyxin molecule and the fatty acyl chains of lipid A [82]. The result of a dynamic simulation study on a laboratory strain of *E. coli* predicted that polymyxin B1 is likely to interact with both the inner and outer membranes via distinct mechanisms. While polymyxin B1 molecules aggregate in the LPS region of the outer membrane with restricted insertion within the lipid A tails, they readily insert into the inner membrane core. The concomitant increased hydration may be responsible for bilayer destabilization and antimicrobial function [83].

A third model of polymyxins’ activity involves free radical-induced death. In bacterial cells, the activity of redox enzymes extracts the electrons from molecular oxygen, continuously forming intracellular superoxide, hydroxyl radical, and hydrogen peroxide. These species adversely affect the activities of enzymes and the integrity of DNA, lipids, and proteins, thereby compelling organisms to protect themselves with a response involving repair systems and enzymes. However, elevated levels of oxidants will eventually poison bacteria [84]. Superoxide levels are postulated to rise when polymyxin molecules enter across the Gram-negative cell wall; superoxide will be enzymatically converted to hydrogen peroxide by cellular superoxide dismutases. Then, hydrogen peroxide will oxidize ferrous iron to ferric iron, forming a hydroxyl free radical. When the concentration of the latter reaches significant levels, it will stimulate oxidative damage of biological molecules with deleterious effects on the cell, which are independent of polymyxin binding to its specific target in the outer membrane [85]. Therefore, it is suggested that some antibiotics, including polymyxin B and colistin, but also others like kanamycin, kill bacteria through oxidative stress and reactive oxygen species (ROS) generation [3]. The applicability of such a mechanism to polymyxin has been investigated in several studies. For instance, sublethal concentrations of polymyxin B induced an oxidative burst and high endogenous ROS production in *P. aeruginosa* [86]. Moreover, a hydroxyl radical scavenging compound, thiourea, was assessed regarding its ability to prevent the polymyxin-induced killing of *A. baumannii*. A striking decrease in the ability of both polymyxin B and colistin to kill *A. baumannii* was noticeable in the presence of thiourea, which acted as a “rescue” compound [87]. In 2015, Dong et al. showed that polymyxin stimulates the generation of ROS, but cell killing could occur by nonoxidative mechanism, typically envelope disruption. Furthermore, polymyxin stimulates the expression of a gene called *soxS* (for superoxide response) in E. coli, whose expression may be a strategy to prepare for the potentially lethal interactions with diffusible toxic molecules, including polymyxin. The gene *soxS* encodes a transcriptional activator of genes that respond to reactive oxidative (redox) stress, such as *sodA* (Mn-containing superoxide dismutase), *fpr* (NADPH:ferredoxin oxidoreductase), and *ydbK* (a putative Fe-S-containing reductase) [88]. Interestingly, in 2017, it was shown that colistin induced ROS accumulation and oxidative stress-induced damage in *P. polymyxa*, the producer of colistin. This highlighted an unfamiliar activity of colistin against Gram-positive bacteria [89]. Moreover, the membrane damage and extensive cell surface alteration in Gram-positive bacteria was revealed by detecting the leakage of intracellular molecules, shedding light on a not yet described bactericidal mechanism of polymyxin E against Gram-positive bacteria [90].

Additional systems of polymyxin activity have been proposed. By binding to the LPS molecule released upon the cell lysis of Gram-negative pathogens, polymyxins cause the neutralization of the endotoxin, corresponding to lipid A of LPS, diminishing its pathophysiologic effects in the circulation [12]. Moreover, Deris et al. showed that polymyxins inhibited key respiratory enzymes, such as type II NADH-quinone oxidoreductases, which exist in the bacterial inner membrane of three Gram-negative species: *E. coli*, *K. pneumoniae* and *A. baumannii*, suggesting this pathway as a secondary polymyxin mode of action [91]. A recent study showed the enhancement of NADH metabolism and the resulting generation of oxidative damages in Gram-positive cells of *Bacillus subtilis* and *P. polymyxa*, giving insights into a yet unrevealed action of polymyxin in Gram-positive cells [92]. Additionally, a significant decrease in dividing cells was observed when *P. aeruginosa* were treated with colistin, although without significant killing [93]. In this report, an increase in cellular rigidity was noticeable, speculating that the binding of colistin to the outer membrane may stiffen the bacterial cell wall, altering its nanomechanical properties and morphology, and perturbing normal cell division. McCoy and colleagues [94] showed that polymyxins can bind to the 16S (prokaryotic) and 18S (eukaryotic) A-site RNA constructs of ribosomes, where they interfere with eukaryotic translation in vitro, but not with bacterial translation. Therefore, a possible mechanism for polymyxins by interference with bacterial ribosomes was investigated, but appears unlikely. Recently, and using a metabolomic approach, elevated glucose levels, and various glyoxylate and glycerolipid metabolic intermediates, were observed in *Mycobacterium tuberculosis* cultured in the presence of colistin. The increase in fatty acid synthesis and cell wall repair postulated that colistin acts by disrupting the cell wall in *M. tuberculosis*, in a manner similar to other bacteria [95]. In *Mycobacterium smegmatis*, polymyxin B inhibited the activity of the alternative NADH dehydrogenase and the malate: quinone oxidoreductase, which are both respiratory enzymes [96]. The last two models perhaps justify interest in the applicability of polymyxin as a potentiator of anti-mycobacterial drugs [97].

Overall, despite the traditional record of polymyxins as affecting bacterial membranes leading to lysis and death, there is substantial work investigating secondary mechanisms. Polymyxins shuffle phospholipids, mediate hydroxyl radical death pathways, counterbalance endotoxins, and affect normal cell reproduction and respiration. It is hoped that a deeper understanding of the mechanisms of action of these compounds will offer better insights into their pharmacological potential and clinical utility. 

## 5. Bacterial Resistance to Polymyxins and Changes in Membranes

The use of polymyxins, among the few remaining valid options for the therapy of infections caused by MDR *Enterobacteriaceae*, *P. aeruginosa* and *A. baumannii*, has prompted the emergence of resistance to this antibiotic class, creating a major public health concern [98]. Bacterial resistance to polymyxins may be chromosomal and associated with the modification of LPS, or may be encoded on transposable genetic elements, namely *mcr* genes [43]. A representation of the current knowledge regarding bacterial resistance to polymyxins is discussed below.

### 5.1. Chromosomal Resistance

Similar to bacterial species that are naturally resistant to polymyxins (described above), the change in the LPS total charge is responsible for developing polymyxin resistance [12]. Chromosomal mutations that lead to the addition of cationic groups to lipid A weaken the binding of polymyxins to their main target [23]. Although the exact chromosomal mechanism for lipid A modification appears to be species-specific, regulatory genes or operons such as PmrAB and PhoPQ trigger the chromosomal mechanism and are shared by many species [99,100]. The role of these regulatory systems is to permit bacterial cells to react to environmental changes by modifying their gene expression. When these regulatory systems interact with one another, they even have more profound effects on polymyxin resistance [101]. The net effect of the activity of these systems is the addition of cationic groups, 4-amino-4-deoxy-L-arabinose (L-Ara4N) and pEtN, to LPS, mediating the acquired resistance to colistin [12]. Below is a panel of operons involved in this process:

1. **The PhoPQ two-component system and its regulatory gene *mgrB*.** This system codes for two proteins, the regulator protein PhoP and the protein kinase *PhoQ*. While the kinase senses a specific environmental stimulus, the corresponding response regulator mediates the cellular response, mostly through the differential expression of target genes. In the presence of certain environmental stimuli, this system allows the expression of virulence factors, enzymes that modify the LPS to allow resistance to cationic antimicrobial peptides, or enzymes that decrease stress due to acidic pH. The PhoPQ two-component system promotes bacterial survival in low magnesium concentration or in acidic pH or in the presence of cationic antimicrobial peptides. *PhoQ* is a protein with tyrosine kinase activity that activates PhoP through phosphorylation [102]. Active PhoP drives the transcription of the *pmrHFIJKLM* operon, involved in the chemical modification of LPS via the addition of L-Ara4N to the LPS. Moreover, PhoP can also activate the pmrA gene, triggering the expression of PmrA protein, causing the addition of pEtN to the LPS [103]. The regulation of the PhoPQ system occurs through the gene *mgrB*, which acts as a negative regulator. Upon the activation of PhoP, the *mgrB* gene is upregulated. The translated *mgrB* protein in turn represses the *PhoQ* gene. The inactivation of the *mgrB* gene leads to the overexpression of the phoPQ operon, thus causing *pmrHFIJKLM* operon activation, leading to the production of L-Ara4N responsible for the acquisition of polymyxin resistance. Studies show that substitutions, insertions, or deletions in the *mgrB* gene mediate polymyxin resistance [12]. For example, in KPC-producing *K. pneumoniae*, the transcriptional upregulation of the *PhoQ* gene was observed in the strains with *mgrB* alterations, mediating colistin resistance [104]. Although *mgrB* mutations or inactivation were suggested as major mechanisms for colistin resistance in *K. pneumoniae* [105,106,107], Borsa et al. reported an overexpression of *PhoQ* and phoP genes in *K. pneumoniae* with wild-type *mgrB* gene, suggesting that other genetic regulations of the PhoPQ system may exist [108]. A very recent report from Korea described the *mgrB* alteration mediating colistin resistance in E. coli isolated from livestock [109]. It is noteworthy that the mutation of genes other than *mgrB* may contribute to enhancing PhoPQ system activity, such as ColR/ColS and CprR/CprS regulatory systems in *P. aeroginosa* [110], and cprR/cprS in *C. jejuni* [99].
2. **The PmrAB two-component system.** Similar to the PhoPQ system, the PmrAB system is a typical two-component system, so it encodes both PmrA and PmrB. PmrB is a protein with tyrosine kinase activity, that activates the transcriptional regulator PmrA by phosphorylation. Environmental stimuli, such as macrophage phagosomes, ferric iron, aluminum ion, and low pH, activate PmrB. PmrA in turn activates the transcription of the *pmrCAB* operon and the *pmrHFIJKLM* operon, that are involved in LPS modification by the addition of pEtN and L-Ara4N [111]. Mutations causing constitutive activation in the *pmrA* and *pmrB* genes have been described as being responsible for acquired colistin resistance [99]. Reports of such alterations are availble for *E. coli* [112], *Enterobacter cloacae* [113], *P. aeruginosa* [114], and *A. baumannii* [115,116].
3. **The *lpxA*, *lpxC* and *lpxD* genes.** This unique set of genes exists in *A. baumannii*, which can become highly resistant to polymyxins via spontaneous mutations in these lipid A biosynthesis genes. If the biosynthetic lipidA genes, *lpxA*, *lpxC*, or *lpxD*, become inactive, LPS is not formed, and interaction with polymyxins is lost [116]. In its attempt to adapt to the antibiotic pressure induced by polymyxins, *A. baumannii*, through the inactivation of the aforementioned genes, loses LPS, a major virulence factor and structural component. Such adaptation results in a dramatic decrease in the fitness and virulence and major changes in the physiology, thus providing insights into the low prevalence of polymyxin-resistant *A. baumannii* isolates with LPS loss in the clinical setting [117].

In addition to the cationic modifications of lipid A, some studies indicated the prevalence of other changes, such as outer membrane remodelling events, which may contribute to resistance. The comparative profiling of the outer membrane proteome of a laboratory strain of extremely colistin-resistant *K. pneumoniae* revealed that outer membrane proteins from bacterial stress response, glutamine degradation, aspartate, pyruvate, and asparagine metabolic pathways were over-represented, compared to sensitive strains [118]. In clinical strains of *K. pneumoniae* and *Enterobacter asburiae*, matrix-assisted laser desorption ionization time of flight/mass spectrometry (MALDI-TOF/MS) investigation done by a Hungarian research group identified the differences between outer membrane proteins among colistin-susceptible and -resistant counterparts. While the colistin-susceptible *K. pneumoniae* had 16 kDa proteins belonging to the LysM domain/BON superfamily, as well as DNA starvation proteins, the colistin-resistant strains had OmpX and OmpW. Furthermore, OmpC and OmpW were detected in the colistin-susceptible *E. asburiae*, whereas OmpA and OmpX were identified in the colistin-resistant counterpart. This demonstrated that the altered Gram-negative cell wall may contribute to acquired colistin resistance in *Enterobacteriaceae* [119]. Another study investigated the influence of lipid A acylation pattern on the crucial interaction between the LPS of a clinical *K. pneumoniae* isolate and polymyxins. The underacylation of lipid A resulted in increased polymyxin susceptibility, with the hexa-acylated lipid A showing better interaction with polymyxins than the penta-acylated lipid A, perhaps unraveling a novel appreciation of the mechanisms of polymyxin activity and resistance [120].

### 5.2. Plasmid-Mediated Resistance

Until 2015, the identified mechanisms of polymyxin resistance were attributed to chromosomal mutations, and not to horizontal gene transfer. However, it was found that polymyxin resistance could also be dependent on plasmid-mediated and therefore the transferable genes, of the mobilized colistin resistance (*mcr*)-type [121]. Such mobile resistance is ultimately rendering the “last resort” polymyxin antibiotics therapeutically unusable, and is disseminating over wide geographic locations, as well as among animals, water, food chain and the environment [122]. 

The first *mcr* gene, *mcr-1*, was identified in the *E. coli* of animal, human, and environmental origin recovered in China in 2015, during the routine surveillance of antimicrobial resistance in *E. coli* from food animals [123]. Being carried on a conjugative plasmid, *mcr-1* exhibited an easily driven dissemination into various bacteria from animals and humans. In light of a such transferability of polymyxin resistance by *mcr-1*, it was not surprising that it rapidly swept across nearly the entire globe in less than a year since its first discovery [124]. Eventually, different variants up to *mcr-9* were identified in various Gram-negative bacteria [43], and were reported beyond China in all continents [12]. In 2020, a novel *mcr* gene, *mcr-10*, was identified on a plasmid of an *Enterobacter roggenkampii* clinical strain [125]. A summarized description of the known *mcr* genes to date is shown in Table 2. The *mcr-1* gene is responsible for polymyxin resistance through encoding a pEtN transferase. This enzyme, similar to chromosomal resistance pathways, especially those associated with two-component systems, mediates the addition of pEtN to lipid A, making this compound highly cationic [126]. In terms of membrane changes, the *mcr* phenotype in Gram-negative bacteria is known to decrease the membrane charge and increase its packing by the chemical modification of the outer membrane. In this sense, the cationic additions reduce the negative charge and limit antimicrobial peptide binding and membrane disruption. These modifications make the outer membrane more resistant to antimicrobial peptides, and suggest that LPS modification prevents the penetration of large molecules through a strengthening of lateral interactions between neighboring LPS molecules. When lipid packing increases, the area-per-lipid in the outer membrane is reduced, and polymyxin penetration drastically decreases, as the drug preferably lies flat on the membrane and does not penetrate the cell. In general, a greater packing of lipids is believed to lower the damage caused by polymyxin, corresponding to resistance [127]. 

Molecular investigations of the genetic background of *mcr* genes have confirmed them on three major types of plasmids: IncI2, IncHI2 and IncX4, in addition to IncHI1, IncF, IncFI, IncFIB, IncFII, IncP, IncP-1, IncK2 and phage-like IncY [7]. Some but not all plasmids carrying the *mcr-1* gene harbor other antimicrobial resistance genes to other antibiotics including β-lactams, aminoglycosides, quinolones, fosfomycin, sulfonamides, and tetracyclines. Hence, the location of this gene on multidrug resistance plasmids is worrisome because using other antimicrobials can selectively promote the growth of isolates carrying *mcr-1* and their subsequent spread [12]. More importantly, the *mcr-1* gene has been identified in highly drug-resistant pathogens harboring plasmids encoding carbapenemase genes, such as *bla*_NDM-1_, *bla*_NDM-5_, *bla*_OXA-48_, *bla*_KPC-2_, and *bla*_VIM-1_, significantly complicating the therapy of infections caused by such bacteria [136,137]. 

The origin and associated sequences of the plasmid-encoded *mcr* remain the subject of intensive investigation, and remarkable progress in knowledge pertaining to these details is notable. In addition to the plasmid types and up to 10 variants of the gene reported so far, the insertion sequence of IS*Apl1* was identified to flank one or both ends of *mcr-1*, suggesting that this gene was mobilized by an IS*Apl1* composite transposon which has, in some cases, subsequently lost one or both copies of IS*Apl1* [138,139]. The transposon Tn*6330* was found to be the key element mediating the translocation of *mcr-1* into various plasmid backbones through the formation of a circular intermediate, and it allows the co-transmission of *mcr-1* with other resistance determinants through IncHI2 plasmids [140]. The genetic structure harboring the gene is known as “*mcr-1* cassette”, that has a length of 2600 bp, and was found to carry its own promoter sequences driving the expression of *mcr-1* [12]. Recently, a comprehensive analysis of all *mcr-1* sequences in GenBank was used to identify a chromosomal region of a novel *Moraxella* species, with significant homology to the *mcr-1* structure, and which likely represents the origin of this gene. All *mcr-1* structures lacking one or both flanking IS*Apl1* were obtained from ancestral composite transposons that subsequently lost the insertion sequences by a process of abortive transposition. The mobilization of *mcr-1* occurs as part of a composite transposon and structures lacking the downstream IS*Apl1* are not capable of gene mobilization [141]. The combination of the IS*Apl1* and the *mcr-1* cassette has been described on the chromosome of an *E. coli* isolate recovered in 2015 from raw chicken meat in Switzerland, suggesting the possible hypothesis that *mcr* genes are capable of integration and therefore stabilization in some bacterial chromosomes [142]. 

Epidemiologic data regarding *mcr* genes are also becoming more accessible. In a recent meta-analysis involving over 200 studies from 47 different countries across six continents [65], the overall prevalence of *mcr* genes ranged between 0.1% and 9.3%, with the highest number of *mcr*-positive strains reported in China. Approximately 95% of *mcr* genes were of the type *mcr-1*. The highest prevalence was in the environment (22%), followed by animals (11%), food (5.4%), and humans (2.5%). Pathogenic *E. coli* (54%), isolated from animals (52%) and harboring an IncI2 plasmid (34%) were the species with highest prevalence of *mcr* genes. The significant role of food chain and/or the environment in *mcr* gene spreading is, therefore, evident and warrants further investigation. Given such a global burden of *mcr* gene carriage, its reservoirs, and its dissemination has triggered extensive worldwide alarms [143]. Large-scale investigations are urgently required for a better understanding of the molecular epidemiology and resistance mechanisms of these genes. The proper understanding of transferable polymyxin resistance shall provide evidence to improve clinical therapeutics targeting severe infections by *mcr*-harboring pathogens [144].

## 6. Special Features and Spread of Polymyxin Resistance among Prominent Gram-Negative Pathogens

The features of polymyxin resistance exhibit some specific distinctions among important Gram-negative pathogens, namely those of the family *Enterobacteriaceae*, *P. aeruginosa* and *A. baumannii*. These special features are differentiated below and summarized in Table 3. 

### 6.1. Enterobacteriaceae

Among the family *Enterobacteriaceae*, polymyxin resistance has been described for many species, including *Escherichia*, *Klebsiella*, *Salmonella*, *Shigella*, *Enterobacter*, and *Citrobacter* [145,146], (Table 3). Several molecular mechanisms have been identified, primarily the modification of LPS through the addition of the cationic groups L-Ara4N and pEtN [99]. Specifically, fine gene alterations are responsible for the LPS modifications seen in members of the *Enterobacteriaceae* family, and they can be variable among the different species. 

As far as *K. pneumoniae* is concerned, it is known that a profound molecular mechanism that leads to the emergence of polymyxin resistance in this organism is the mutation/inactivation of the *mgrB* gene, as described above in Section 5.1 [104]. The gene *mgrB* is a conserved gene of 141 nucleotides in length, encoding a short, 47-amino acid protein that causes negative feedback on the PhoPQ regulatory system. Another proposed mechanism that may occur on *K. pneumoniae* is the shedding of capsular polysaccharides from its capsulated surface [148,170]. These capsular compounds have the capability of binding to or trapping polymyxins, thereby depleting drug concentrations reaching the bacterial membranes, and increasing resistance. The proposed binding is a function of the electrostatic interactions between the cationic polymyxins and anionic capsular polysaccharides [171]. Additionally, in a report from India, it was found that a mutant form of the efflux pump KpnEF may increase sensitivity to colistin in *K. pneumoniae*. This pump belongs to the small multidrug resistance (SMR) protein family and is composed of four transmembrane alpha-helices that span the outer membrane and the cytoplasmic membrane. This finding raises the probability that the efflux pump overexpression may contribute to colistin resistance in this organism (Table 3) [149].

In *E. coli*, and parallel to the *mgrB* mechanism, *mgrR* is a key genetic determinant of polymyxin resistance in this organism [63]. The *mgrR* mediates the modification of the outer Kdo (3-deoxy-D-manno-octulosonic acid) residues of LPS by adding pEtN [152]. In *Salmonella*, the gene *mig-14* has been recently shown to contribute to polymyxin resistance through the decreasing permeability of the organism’s outer membrane and endorsing biofilm formation. The reduction in permeability is thought to occur through the ability of *mig-14* to suppress or inhibit the expression of outer membrane proteins OmpF and/or OmpC [157].

Apart from the above chromosomal gene alterations, *Enterobacteriaceae* were the first reservoir of the transferrable polymyxin resistance, with the plasmid-encoded gene *mcr-1* being first reported in November 2015 from *E. coli* [123]. Horizontal spread accounted for the easy dissemination of *mcr* genes among other members of the family. To date, *E. coli* remains the most prevalent species among the *mcr*-positive isolates, accounting over 90% of the total *mcr*-carrying isolates, followed by *Salmonella enterica* (7%) then *K. pneumoniae* (2%). As of 2019, *mcr* has been detected in *Enterobacteriaceae* in 47 different countries [7]. Animals are believed to be the main reservoir of *mcr*, and this is attributed in part to the heavy use of polymyxins in veterinary medicine for prophylactic purposes, and in part due to genetic background. These genes are often associated with *bla*_CMY-2_ and *florR* genes, which are found in animal enterobacterial isolates, and also with an insertion sequence, IS*Apl1*, known in *Pasteurella multocida*, an animal pathogen [172]. Nowadays, *Enterobacteriaceae* harboring *mcr* genes are recovered from patients and healthy subjects [173], hospital surfaces [174], raw meat [123], livestock [175], fresh vegetables [176], wild birds [177], and surface samples from public transportation [178], indicating community spread. Moreover, *mcr* genes are being detected in enterobacterial isolates with other resistance genes. In Tunisia, *E. coli* isolates with both CTX-M-15 and mcr-1 were recovered from bovine fecal samples and raw goat milk [179]. In Brazil, the emergence of a *K. pneumoniae* isolate with both *bla*_KPC_ and *mcr-1* was reported [180]; likewise, the same gene combination was found in *Enterobacteriaceae* growing in streams and wastewater treatment plants in Italy [181]. It is noteworthy to mention that *mcr-1* was detected in Switzerland on the chromosome of an *E. coli* isolate recovered from chicken [142], suggesting that it may be integrated and stabilized in an enterobacterial genome.

Worldwide, the rates of polymyxin resistance in *Enterobacteriaceae* are widely variable. In *K. pneumoniae*, it reaches about 9% worldwide, while in some European countries such as Italy, Greece, Spain, and Hungary, it may rise up to 43%, 20.8% in Greece and over 30% in Spain [4]. In Dubai, the resistance of *Enterobacteriaceae* isolates to colistin was 27% [182]. Considering the further limitations of the antimicrobial options available for the management of infections caused by MDR *Enterobacteriaceae*, there is no doubt that the recent reports of polymyxin resistance in these pathogens raises major concerns, and stresses the need for better surveillance and infection control.

### 6.2. Pseudomonas aeruginosa

*P. aeruginosa* is a Gram-negative opportunistic pathogen of hospitalized and immunocompromised patients, causing severe infections and representing a challenge to infection control. Polymyxins represent the last antibiotic option for *P. aeruginosa* infections [183]. Five two-component systems that regulate LPS modifications are known to mediate polymyxin resistance in *P. aeruginosa* (Table 3) [12]. Much alike has been seen in *Enterobacteriaceae*, where alterations in the PmrAB [160,184,185] and PhoPQ [159] systems have been shown to intervene in acquired resistance to colistin. One report indicated that there is a differential role for these systems, where resistance was caused by both the inactivation and/or amino acid substitutions in *PhoQ*, while resistance was caused only by amino acid substitutions of PmrB. Meanwhile, the alteration of both *PhoQ* and PmrB resulted in higher levels of resistance than the alteration of either component alone [186]. 

The remaining three types of two-component systems shown to participate in colistin resistance in *P. aeruginosa*, are the CprRS [161], and ColRS [110] ParRS [162] systems. Mutations in the ColRS and CprRS two-component regulatory systems may play a major role, since the association of mutations in the *PhoQ* gene and mutations in the *colS* or *cprS* genes confers a high level of polymyxin resistance [110]. The action of the ColRS and CprRS systems may occur through the activation of *PhoQ* gene and/or through other genes that are yet to be identified [12]. Furthermore, the ParRS (polymyxin adaptive resistance) two-component system is involved in adaptative resistance to polymyxins. Mutations in this system cause the constitutive expression of *pmrHFIJKLM* operon with a resulting addition of L-Ara4N to the LPS [187]. 

In addition to the above mechanisms, and very recently, Puja and colleagues showed that the efflux pump MexXY/OprM was important in the adaptation of *P. aeruginosa* to polymyxins, unlocking a new perspective for restoring its susceptibility by the suggested use of efflux inhibitors [163]. In an experimental simulation of infections, where *P. aeruginosa* cells were cultured in the presence of cell wash fluids containing LPS, cell-free LPS derived from bacterial cells inhibited the antimicrobial activity of colistin. This indicated that large amounts of broken and dead cells of *P. aeruginosa* at infection sites may reduce colistin effectiveness, even in cells that have not yet acquired resistance [188]. Moreover, an investigation using lipidomics and transcriptomics discovered that polymyxin B induces lipid A deacylation in *P. aeruginosa.* This mechanism is considered an “innate immunity” response to polymyxins and a compensatory mechanism to L-Ara4N modification, and thus high-level polymyxin resistance in *P. aeruginosa*. The less hydrophobic lipid A with five acyl residues decreased polymyxin B penetration, compared to the normal form with six residues instead [164].

Transferable polymyxin resistance, represented by the spread of *mcr* genes, has been recently rising in *P. aeruginosa*, following its initial observation in *Enterobacteriaceae*. Perhaps the first report of *mcr* in *P. aeruginosa* originated from Maryland, USA, in 2018, when Snesrud and Colleagues, through whole-genome sequencing, discovered a chromosomally encoded *mcr-5* gene carried within a transposon in a colistin-nonsusceptible *P. aeruginosa* [165]. Following that report, another single isolate of *P. aeruginosa* harboring *mcr-1* was detected during a multihospital survey on polymyxin resistance in Pakistan in 2019 [166]. The origin, transmissibility, and prevalence of *mcr* genes in *P. aeruginosa* remains to be further identified.

The spread of *P. aeruginosa* resistant to polymyxins is variable worldwide. In a survey from Taiwan, the rate of resistance was close to 9% [189], which was about 6% in a German university hospital [190], 5% in Spain [191], 4% in Korea [192], and 2% in Thailand [193]. In a cross-sectional, multicenter survey from Dubai, all the studied *P. aeruginosa* isolates were colistin sensitive [194]. On the other hand, the rate of resistance was 22% in a report from India [195], and 15% in a nationwide Italian survey [196]. Ongoing surveillance is needed on a global scale, to observe and mitigate the spread of polymyxin resistance in this hostile pathogen.

### 6.3. Acinetobacter baumannii

Nosocomial infections with *A. baumannii* constitute a global problem, with high mortality rates and resistance to most antibiotics [197]. The rapid emergence of resistance in this top priority pathogen has revived clinical interest in polymyxins. However, resistance to this class of antibiotics in *A. baumannii* is on the rise [116]. Although the current rate of polymyxin-resistant *A. baumannii* represents less than 1% of clinical isolates, these still pose a significant challenge to public health authorities [198].

The main mechanism of polymyxins resistance in *A. baumannii* is chromosomally encoded, and relies on spontaneous mutations in lipid A biosynthesis genes, *lpxA*, *lpxC*, or *lpxD*, where LPS is not formed, and interaction with polymyxins is lost [199]. Mutations detected in those genes were either substitutions, truncations, frameshifts, or insertional inactivation by the insertion sequence IS*Aba11* [200]. Moreover, the addition of cationic groups to the LPS can occur in *A. baumannii* and is mediated by mutations in PmrAB [201]. On another note, the overexpression of genes corresponding to reduced fluidity and the increased osmotic resistance of the outer membrane, known as *lpsB*, *lptD*, and *vacJ*, was shown to contribute to polymyxins’ resistance in *A. baumannii* [202]. In a strain of *A. baumannii* with laboratory-induced polymyxin resistance, the expression of the efflux pump EmrAB was found to be high, validating the possible association between EmrAB efflux pumps and the decreased sensitivity to polymyxins in *A. baumannii*. Nevertheless, the clinical implications of this association remain to be tested [168]. In 2018, a study from Australia showed that an extra copy of the insertion sequence element IS*Aba125* within a gene encoding an H-NS family transcriptional regulator of *A. baumannii*, contributed to pEtN transferase and colistin resistance [203].

The conventional, chromosomally encoded resistance to polymyxins in *A. baumannii* has a limited spread. However, the plasmid-borne *mcr* gene has been recently described in *A. baumannii*. There have recently been reports of *mcr-4* from the Czech Republic [204], *mcr-1* from Pakistan [166], as well as *mcr-4.3* from China [169] and Brazil [205]. The explication of genotypic profiles and resistance mechanisms are essential to control resistance to polymyxins in *A. baumannii*, thereby preserving this antibiotic class as a treatment option.

## 7. Future Implications

With the necessity of polymyxins for the management of MDR infections, it is anticipated, like what has been seen with other classes of antibiotics, novel generation polymyxins will be sought. Therefore, improving the properties of the two clinically available polymyxins shall be of prodigious interest to researchers focusing on antimicrobial development. Over the last decade, and in light of the augmented research on polymyxin pharmacology, the mode of action and toxicity, as well as the chemical and molecular elucidation of resistance, investigations have yielded a number of promising pathways to boost polymyxins efficacy and safety and reduce bacterial resistance [8]. 

For example, and in terms of medicinal chemistry, it was discovered that the incorporation of amino acid residues with long lipophilic alkyl or biphenyl side chains, such as octyl-glycine and biphenylalanine, at positions 6 or 7 of the polymyxin molecule, resulting in better activity against resistant isolates [206]. The observation that the N-terminal fatty acyl chain represents a major cause for nephrotoxicity, has directed chemical modification efforts to re-construct polymyxins by focusing not only on the N-terminal fatty acyl group, but also to look beyond the peripheries of the polymyxin scaffold and examine all the amino acid residues within the polymyxin core structure. This has given rise to exciting novel compounds, which should be further refined to structurally uncouple the antimicrobial activity from nephrotoxicity [25]. 

Other efforts are focusing on modified polymyxin formulations or synergistic combinations that decrease toxicity; Yu and colleagues have recently developed an inhalable liposomal mixture of colistin and ciprofloxacin, that showed high effectiveness against *P. aeruginosa* [207]. Other interesting formulations of polymyxins are structured gels based on natural and synthetic polymers [208]. For example, phenylboronic acid polycarbonate hydrogels, that are loaded with polymyxin B, demonstrated in vitro antimicrobial efficacy against *P. aeruginosa* from burn wound infections [209]. The use of microneedles for the transdermal delivery of drugs into the systemic circulation was also investigated for polymyxins. A microneedle system composed of sugar, polyvinylpyrrolidone and encapsulated polymyxin B, was developed and used against *Salmonella typhimurium*, with the achievement of a high antibiotic level in models of porcine skin [210,211]. Innovative delivery systems of polymyxins include microparticles, nanoparticles, liposomes, and niosomes [208]. A niosome is a non-ionic vesicle comprising a non-ionic surfactant and cholesterol, and can incorporate both hydrophilic drugs (in its aqueous layer) and lipophilic drugs (in its vesicular lipid membrane) [212]. Niosomes can enhance the delivery of poorly absorbable drugs and increase drug absorption and bioavailability by the penetration of the GIT barrier. Polymyxin B niosomes were formulated using sorbitan monostearate surfactant and cholesterol to improve intestinal permeability. They protected polymyxin B from the GIT environment and increased its absorption within the normal limits of nephrotoxicity, indicating that niosomes improve the oral bioavailability of polymyxin B. There is no increase in the side effects with this mode of delivery [213]. Therefore, the improved delivery and actions of polymyxins shall be obtained by a blend of chemistry, nanotechnology, and pharmaceutics.

The recent trending strategy in testing FDA-approved medications that have no antibacterial activity in combination with antibacterials to enhance the latter’s effect is being applied to polymyxins. In a truly ground-breaking approach, researchers from Australia found that a combination of polymyxin B and the selective serotonin reuptake inhibitor, sertraline, resulted in greater damage to *P. aeruginosa*, highlighting the likely possibilities of this combination for the treatment of central nervous system infections [214]. Moreover, the experimental antitumor compound PFK-158 was found to enhance the activity of colistin and delay resistance emergence in mice infected with *Enterobacteriaceae* [215]. In clinical trials, colistin showed synergistic activity with the antiviral drug azidothymidine in the management of urinary tract infections caused by *mcr-1*-producing *E. coli* [216]. A trial combining polymyxin B with the estrogen modulator tamoxifen detected a remarkable decrease in the essential precursor metabolites of L-Ara4N, interfering with a major mechanism of polymyxin resistance. The combination had a synergistic bactericidal effect on polymyxin-resistant *P. aeruginosa* in a cystic fibrosis metabolomic model [217]. 

Apart from such an approach, and considering the research on antimicrobial peptides other than polymyxins, in a study testing experimental cationic antimicrobial peptides, Geitani and colleagues [218] showed that the minimum inhibitory concentrations of colistin on *P. aeruginosa* were reduced by eight-fold in the presence of these peptides, probably suggesting an interesting alternative combination for resistant strains. Moreover, in a Turkish investigation on *P. aeruginosa* biofilms that were initially resistant to therapeutic concentrations of antibiotics, colistin was synergistic with antimicrobial peptides for inhibiting the attachment of bacterial cells to a biofilm surface as well as inhibiting biofilm formation [219]. Such examples may prove useful in future attempts to combine polymyxins with antimicrobial peptides as an interesting alternative mode of treatment of MDR pathogens.

In brief, industry and research groups across the globe are still gearing up to develop new polymyxins that are safer and more effective than the currently approved polymyxin B and colistin. For instance, avoiding direct modifications to the polymyxin scaffold while attempting to mimic the physicochemical properties of polymyxins resulted in a series of cyclic amphipathic peptides consisting of alternating cationic and nonpolar amino acid residues, loosely based on the amphipathic properties of polymyxins. These compounds displayed potent antimicrobial activity and a high affinity for LPS [220]. A study by a group from the U.K. and U.S.A. recently reported a series of promising next generation polymyxin nonapeptides with an amine-containing N-terminal moiety of specific regional conformation and stereochemistry. These compounds demonstrated superior in vitro activity and inferior cytotoxicity compared to polymyxin B. A subgroup of these compounds having a β-branched aminobutyrate N-terminus with an aryl substituent also offered low cytotoxicity, and candidates were selected for additional development [221]. The future holds potential for these drug discovery and development programs to bring upgraded polymyxins or novel polymyxin-including combinations from the bench into the clinic.

## 8. Conclusions

To date, significant progress has been made in understanding the activity of polymyxins and unraveling bacterial resistance mechanisms to this refreshed category of antibacterial compounds. However, additional investigations are still underway to obtain a better understanding of membrane interactions, bacterial resistance, and improved clinical utility. In light of increasing resistance to polymyxins, there is a critical need for restricting their use, as well as for effective infection prevention and control measures. Integrated efforts should aim at the robust enquiry of the molecular epidemiology of resistance, and the surveillance of rates and dissemination of resistance among humans, animals, and the environment. The spread of the plasmid-mediated *mcr-1* resistance gene is a principal culprit to polymyxins, which, if unlimited, the real devastating loss of a life-saver antibiotic class may happen soon.

## Figures and Tables

**Table 1:** Minimum inhibitory concentration (MIC) breakpoints of colistin sensitivity according to the European Committee on Antimicrobial Susceptibility Testing (EUCAST) and of colistin/polymyxin B sensitivity according to Clinical Laboratory and Standards Institute (CLSI).

	EUCAST Breakpoints (mg/L)		CLSI Breakpoints	
	S≤	R>	I≤	R≥
*Enterobacteriaceae*	2	2	2	4
*Pseudomonas*	2	2	2	4
*Acinetobacter*	2	2	2	4

S = sensitive; R = resistant; I = Intermediate.

**Table 2:** The 10 discovered variants of the mobilized colistin resistance (*mcr*) gene, with their species and country of first detection, as well as their sequence homology to *mcr-1*.

*mcr* Gene Type	Species of First Detection	Country of First Detection	Sequence Homology to *mcr-1* (%)	Strain Information	Reference
*mcr-1*	*Escherichia coli*	China	100	SHP45	[123]
*mcr-2*	*E. coli*	Belgium	76.7	CP011374	[128]
*mcr-3*	*E. coli*	China	45	WJ1	[129]
*mcr-4*	*Salmonella enterica* serovar Typhimurium	Italy	34	R3445	[130]
*mcr-5*	*S. enterica* subsp. *enterica*	Germany	63.89	11-00422	[131]
*mcr-6*	*Moraxella pluranimalium*	Great Britain	62	248-01T/DSM-22804)	[132]
*mcr-7*	*Klebsiella pneumoniae*	China	65	SC20141012	[133]
*mcr-8*	*K. pneumoniae*	China	31.08	KP91	[134]
*mcr-9*	*S. enterica* subsp. *enterica*	New York	63	GCF_002091095.1	[135]
*mcr-10*	*Enterobacter roggenkampii*	China	29.31	090065 (WCHER090065)	[125]

**Table 3:** Examples of chromosomal and plasmid-encoded polymyxin resistance mechanisms described in *Enterobacteriaceae*, *Pseudomonas*, and *Acinetobacter.*

		Chromosomal Resistance		Plasmid-Encoded Resistance	
		Two-component systems [ref]	Additional mechanisms [ref]	*mcr* type	Ref
*Enterobacteriaceae*	*Klebsiella pneumoniae*	PhoPQ [103] PmrAB [147]	Shedding of capsular polysaccharide capable of trapping polymyxins [148] Overexpression of the efflux pump kpnEF [149]	*mcr-1*	[108]
				*mcr-2*	[108,150]
				*mcr-7*	[133]
				*mcr-8*	[134]
	*E. coli*	PhoPQ [151] PmrAB [112]	Modification of Kdo (3-deoxy-D-manno-octulosonic acid) [152]	*mcr-1*	[123]
				*mcr-2*	[153]
				*mcr-3*	[129]
				*mcr-4*	[154]
				*mcr-5*	[154]
	*Salmonella*	PhoPQ [155] PmrAB [156]	Inhibition of expression of outer membrane proteins OmpF and/or OmpC [157]	*mcr-4*	[130]
				*mcr-5*	[158]
*Pseudomonas*		PhoPQ [159] PmrAB [160] CprRS [161] ColRS [110] ParRS [162]	Efflux pump MexXY/OprM [163] Lipid A diacylation [164] Chromosomal *mcr-5* [165]	*mcr-1*	[166]
*Acinetobacter*		PmrAB [167]	Expression of the efflux pump EmrAB [168]	*mcr-1*	[166]
				*mcr-4.3*	[169]

## Abbreviations

CLSI	Clinical Laboratory and Standards Institute
CMS	colistimethate sodium
Dab	L-α,γ-diaminobutyric acid
EUCAST	European Committee on Antimicrobial Susceptibility Testing
Kdo	3-deoxy-D-manno-octulosonic acid
L-Ara4N	4-amino-4-deoxy-L-arabinose
Leu	leucine
LPS	lipopolysaccharide
MALDI-TOF/MS	matrix-assisted laser desorption ionization time of flight/mass spectrometry
mcr	mobilized colistin resistance
MDR	multi-drug resistant
MIC	mimimum inhibitory concentration
pEtN	phosphoethanolamine
ROS	reactive oxygen species
SMR	small multidrug resistance
Thr	threonine
